# Relationship between impact characteristics and launch direction in softball hitting: A study involving elite players

**DOI:** 10.1371/journal.pone.0260520

**Published:** 2021-11-30

**Authors:** Shuji Kidokoro, Yoshitaka Morishita

**Affiliations:** Department of Sports Research, Japan Institute of Sport Sciences, Kita-ku, Tokyo, Japan; University of Innsbruck, AUSTRIA

## Abstract

In the game of softball, the batter should possess the necessary skills to hit the ball toward various directions with high initial speed. However, because various factors influence each other, there are limitations to the range that can be controlled by the batter’s skill. This study was aimed at extracting the impact characteristics associated with the launch speed/direction and batted ball spin and clarifying the important skills required to improve the batter’s hitting performance. In our experiments, 20 female softball players, who are members of the Japan women’s national softball team, hit balls launched from a pitching machine. The movements of the ball and bat before, during, or after the impact were recorded using a motion capture system. Stepwise multiple regression analysis was performed to extract factors relating the side spin rate. The undercut angle (elevation angle between the bat’s trajectory and the common normal between the ball and bat: ΔR^2^ = 0.560) and the horizontal bat angle (azimuth of bat’s long axis at ball impact: ΔR^2^ = 0.299) were strongly associated with the side spin rate (total R^2^ = 0.893, *p* < 0.001). The undercut angle in opposite-field hitting was significantly larger than that in pull-side hitting (*p* < 0.001). The side spin rate was associated with the undercut angle because the bat’s distal (barrel) side inclined downward (–29.6 ± 8.7°) at impact. The ball exit velocity was higher when it was hit at a smaller undercut angle (R^2^ = 0.523, *p* < 0.001). Therefore, it is deemed desirable to focus on maximizing the ball exit velocity rather than ball spin because the ball–bat impact characteristics vary inevitably depending on the launch direction. Meanwhile, the use of the ball delivery machine and the slower pitched ball are the limiting factors in the generalization of the findings.

## Introduction

In ball sports, the ball can travel at high speeds because of actions such as throwing and hitting. Although ball speed is one of the factors used to evaluate the ability of performance in sports, such as baseball, cricket, and softball [1, 2], ball spin has been considered in some cases when investigating player performance [3–5]. Many baseball studies focusing on ball spin have verified the pitched ball’s flight trajectory or the pitching motion that generates spin [6–9]. However, only a few studies have reported the relationship between the spin rate and travel distance of the batted ball [10]. Furthermore, even if the batted ball launches in a trajectory such that the batter can make a homerun, a higher side spin caused by a vertical tilt of the spin axis may shorten the flight distance. Therefore, understanding the impact characteristics that affect the trajectory of the batted ball, especially the side spin rate, will help improve the offensive and defensive performance.

**Fig 1:**
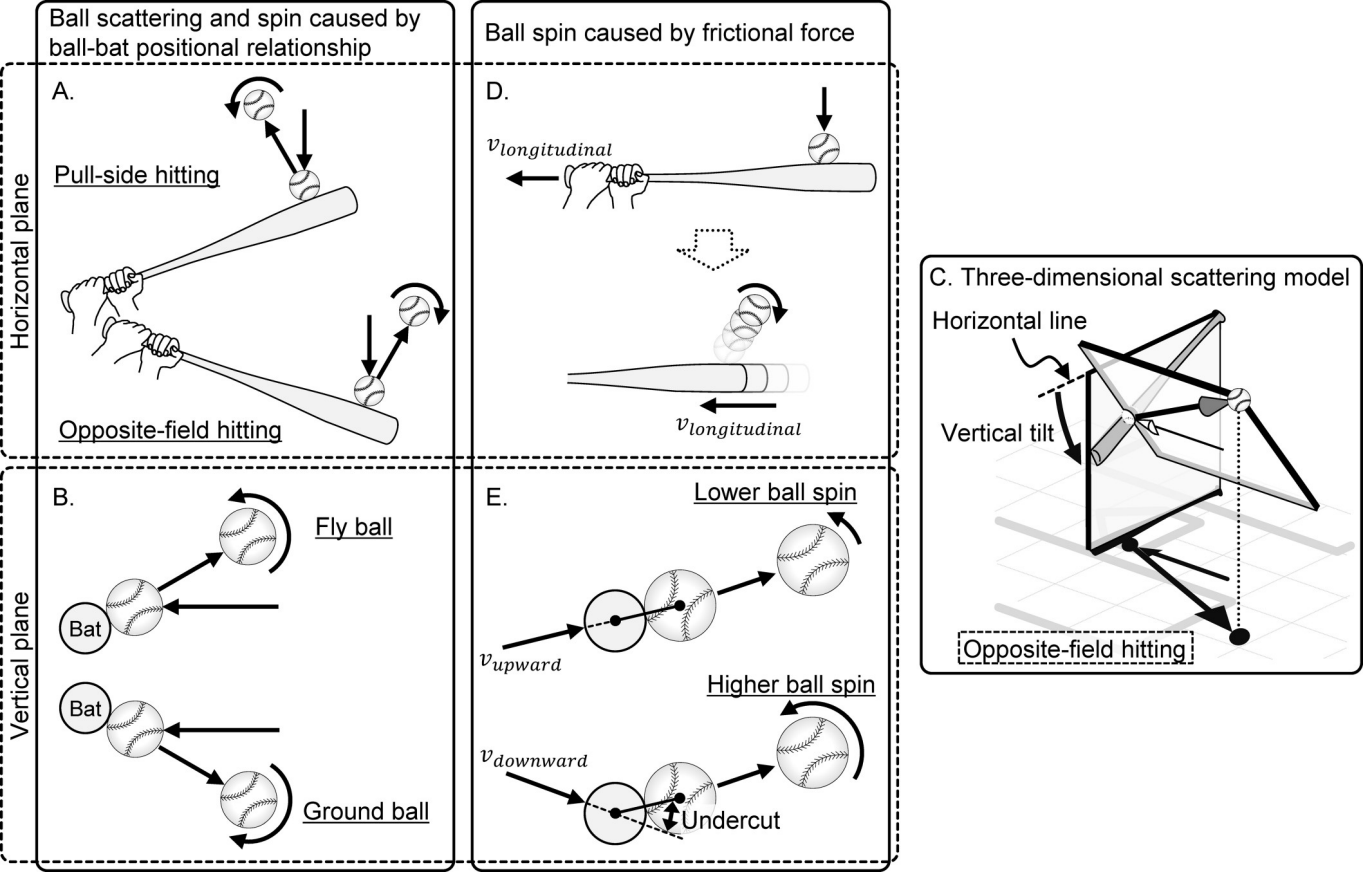
Model of the factors affecting the launch and spin directions of the batted ball. (A) Oblique impact on the horizontal plane. (B) Oblique impact along the short axis cross-section of the bat. (C) Oblique impact when the distal side of the bat is tilted downward. (D) Side spin generation because of the bat velocity toward the proximal side. (E) Backspin or topspin generation because of the upward or downward path of the bat velocity.

A batted ball spins primarily because of the oblique impact of the ball with the bat [11–14]. Scattering the ball in the vertical and horizontal directions because of an oblique impact has been separately verified in two dimensions, namely, horizontal plane (Fig 1A) and vertical plane (Fig 1B), based on the shape and rebound characteristics of the bat [15–18]. The horizontal plane shows the oblique impact caused by the orientation of the bat’s long axis at ball impact, whereas the vertical plane shows the oblique impact along the cross-section of the bat’s short axis. It is important to identify the accurate ball (or shuttle)–bat (or racket) impact location because the bounce characteristics such as the post impact ball speed and angle are changed according to the impact location, and this does not apply only to baseball and softball [19, 20].

In actual bat swings, however, the distal (barrel) end of the bat at the ball impact lies at a lower position than the proximal (knob) end [21, 22]. Therefore, the scattering plane itself fixed to the bat also tilts (Fig 1C). Consequently, even if the impacting surface of the bat is directed toward the center field at ball impact, a fly ball may be directed toward the opposite field [23]. Therefore, baseball hitting is not a bilaterally symmetric motion, and the characteristics of a batted ball will also differ between the pull-side (same-field) and opposite-field hitting. As an example, a study focusing on the batted ball spin reported that when the ball is hit toward the opposite field, the absolute value of the side spin rate is greater on average than that in the pull side [24]. Furthermore, the spin of the batted ball can also be changed by the frictional force resulting from the velocity vector of the bat immediately before impact (Fig 1D and 1E). As mentioned above, many factors change the characteristics of a batted ball; however, to the authors’ knowledge no study has investigated the interaction between the ball and the bat based on the actual bat swing performed by the baseball or softball player. Therefore, the technical factors that substantially affect a ball’s spin rate remain unclear.

The spin rate of a batted ball fluctuates with the change in output variables such as the initial speed and the launch angle of the batted ball [12, 14, 16, 18]. On the other hand, even if the two-dimensionally simulated study, which is mentioned above, can accurately evaluate the interaction between the ball and the bat, it did not include the actual measurement value by the batters’ swing, and thus, the extent to which the findings can be generalized has not been clarified. By understanding the range of variations in the batted ball that can be controlled by the batter’s skill, the technical issue that the batter should focus on can be defined. Therefore, the purposes of this study were to clarify the association between the ball-bat interaction by actual batters’ swing and launch direction of the batted ball in softball hitting. We hypothesized that the fly ball that is directed toward the opposite field includes a “slice spin component,” and it deflects toward the foul ground side regardless of the batter’s technique, because the ball–bat collision characteristics are strongly associated with the launch direction.

## Materials and methods

### Participants

Twenty female softball players, who are members of Japan’s national softball team, participated in this study. All position players, including the two minors who convened in the national team’s training camp, were recruited as participants. The mean ± standard deviation (SD) values of the body height, body mass, and age were 163.2 ± 4.4 cm, 63.5 ± 5.8 kg, and 25 ± 4 years, respectively. No participants had any problems for health condition, and they were capable of performing the swing safely and with maximal effort.

This study was approved by the Ethical Committee of the Japan Institute of Sports Sciences are affiliated before the experiment was conducted. All participants were explained in advance that the purpose, the experimental procedure, the risks, and participation in the experiment was voluntary. After that, they provided written consent to participate in this study. For minors, informed consent was provided by participants and their guardians (i.e., legal representatives).

### Experimental procedure

The experiments were conducted in 12 m (width) × 30 m (depth) × 8 m (height) cages within an indoor experiment room. A spring-type portable pitching machine (L60111J, Louisville Slugger, Louisville, KY, USA) was set up at a position 7.5 m away from the home plate toward the pitcher’s plate. The ball was set to be pitched toward the center of each participant’s strike zone, whether right-handed or left-handed batter. The ball speed immediately before impact was 9.6 ± 0.2 m·s^-1^ (8.7–10.3 m·s^-1^), and it reached the impact point at a downward angle of 19.5 ± 1.8° (11.4–25.2°) from the horizontal direction. The ball coordinates at impact were at -0.001 ± 0.077 m (-0.210–0.261 m) on the right and left relative to the center of the home plate and at a height of 0.723 ± 0.142 m (0.334–1.136 m) from the ground level. Each variable indicates mean ± SD (min.–max.). The pitching conditions were decidedly different from the real game in distance and speed, but because the participants cannot perform the timing control using the visual information of the pitching motion, the conditions for pitching were set to a speed at which all participants could perform their usual swing movements without any problems by observing the timing of ball launch and the launched ball trajectory.

After sufficient warm-up, first, each participant practiced hitting the ball thrown from the pitching machine to familiarize herself with hitting attempt. After that, each participant was asked to hit the ball under three conditions: 1) 10 free hitting attempts with no special restrictions, 2) 10 hitting attempts with the intention of pull-side hitting, and 3) 10 hitting attempts with the intention of opposite-field hitting. Thus, each participant had a total of 30 hitting attempts. To improve the efficiency of the measurement, the order of the three conditions was not randomized; each participant was given 10 free hitting attempts. However, to eliminate the bias of the bat swing as much as possible, the participants were instructed to hit the ball toward each direction alternately after every five attempts. To maintain swing form, participants were asked to prioritize swinging hard against the pitched ball over trying to hit the ball in the intended direction. However, for bad balls and balls that were likely to off-balance their swing form, they took a look the ball based on their own judgment. There were no swing and a miss or obvious missed shots like a foul tip, and in addition, the measurements ended with 30 swings for each participant, regardless of the actual launch direction of the batted ball. The three conditions were established to acquire the data of various launch directions and not to compare conditions.

Approved leather softballs (2OS-15000, Mizuno Corporation, Japan) were used in this study. Each participant was asked to select their preferred bat from two types of fiber–reinforced plastic (FRP) bats with similar characteristics (1CJFS10384, Mizuno Corporation, Japan; and WTLJKS17X, Wilson Sporting Goods Company, Chicago, IL). The two bats possess similar physical specifications: length = 84 cm; diameter of the distal side = 5.7 cm; mass = 700 g; and distance between the proximal end to the center of mass = 51.2 cm. Some players used different bats from ones that they daily use, but the difference in characteristics between the FRP softball bats was small, and thus, players were able to perform their swing with maximal effort without any problems. Therefore, the effect of bat differences on athlete performance is considered to be negligibly small.

### Data analysis

**Fig 2:**
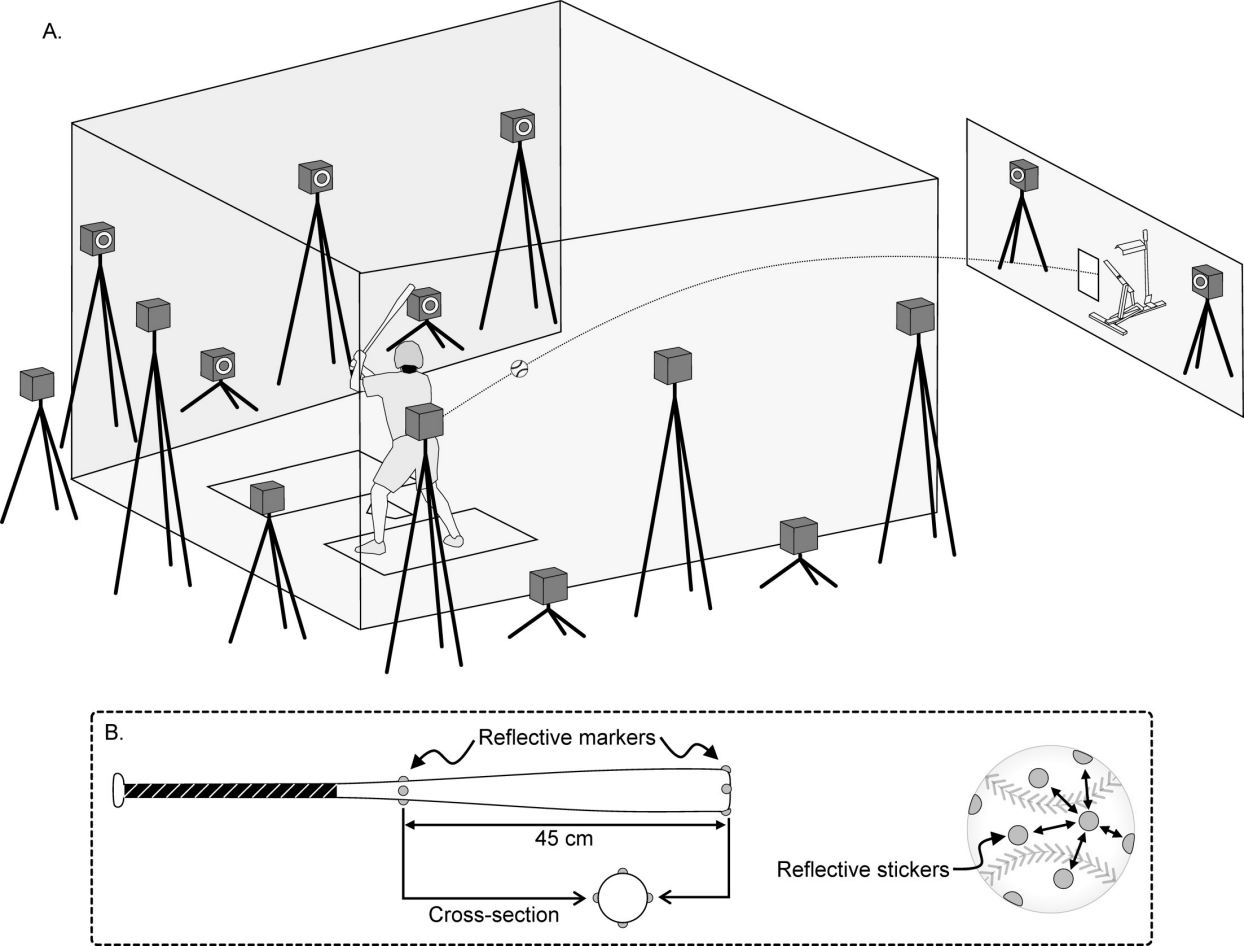
Experimental setup. (A) Schematic of the position of 15 optical cameras. (B) Locations of reflective markers (stickers).

The motions of the ball and bat were recorded using an optical motion capture system (VICON MX, Vicon Motion Systems, Ltd., Oxford, UK). Fifteen synchronized infrared cameras were installed to surround the batter (Fig 2A); the sampling rate was 500 Hz. To obtain the three-dimensional (3D) coordinates of the ball, circular reflective stickers (diameter: 16 mm) were attached at 12 positions on the ball surface. Each circular reflective sticker was attached such that the camera could recognize each sticker as a different marker (Fig 2B). Hemispherical markers (diameter: 10 mm) were attached at four positions on the distal end of the bat so that the midpoint of the marker is the center of the cross-section. Similarly, hemispherical markers were attached at four positions on 45 cm toward the proximal end from the distal end on the bat.

An orthogonal coordinate system was defined with the origin being at the rear edge of the home plate. The direction from the right-handed batter’s box to the left-handed batter’s box was defined as the x-axis, the direction from the home plate toward the pitcher’s plate as the y-axis, and the upward direction in the vertical direction as the z-axis. To describe all data as pertaining to right-handed batters, the positive/negative signs of the x-coordinate values of the left-handed batters were reversed.

**Fig 3:**
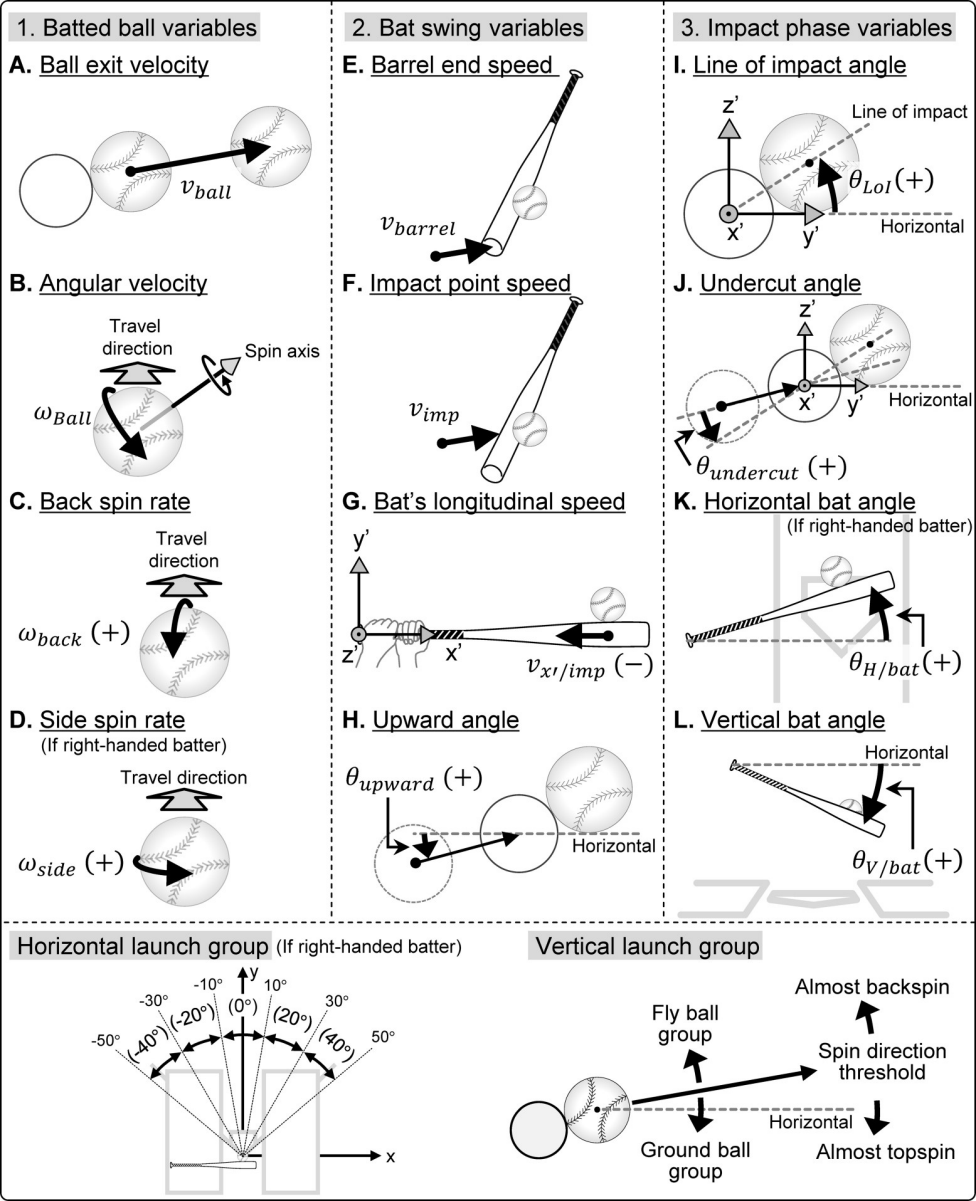
Definition of each variable.

To calculate the position and velocity (speed and direction) of the ball immediately before and after the impact, we estimated the 3D coordinates of the ball center. The center of the ball was estimated from the coordinate values of more than four reflective stickers attached to the surface of the ball by the least squares method using the general equation of a sphere. The definitions and abbreviations of the main variables used in this study are listed in Table 1 and Fig 3.

**Table 1:** Definition and description of parameters in ball−bat interaction.

Symbol		Description	Definition	
**Post-impact ball variables**				
	*v*_*ball*_	Ball exit velocity		Ball speed immediately after impact.
	*θ*_*H*/*ball*_	Horizontal launch angle		Angle between the ball velocity vector immediately after impact projected onto the horizontal plane and the center line. The positive direction is the right field (opposite field) side.
	*θ*_*V*/*ball*_	Vertical launch angle		Angle between the ball velocity vector immediately after impact projected onto the vertical plane and the horizontal plane.
	*ω*_*ball*_	Angular velocity		Post-impact ball angular velocity (resultant angular velocity).
	*ω*_*back*_	Back spin component		Back spin component of the post-impact ball angular velocity.
	ω_*side*_	Side spin component		Side spin component of the post-impact ball angular velocity. The positive direction is “hook” spin.
**Pre-impact bat variables**				
	*v*_*barell*_	Distal (Barrel) end speed		Speed of the distal end of bat immediately before impact.
	*v*_*imp*_	Impact point speed		Speed of the impact point of bat immediately before impact.
	*v*_*x*′*/imp*_	Bat’s longitudinal speed		Speed in the x′-component (along the bat’s long axis) of the impact point immediately before impact.
	*θ*_*upward*_	Upward angle		Elevation angle between the bat’s trajectory immediately before impact and the horizontal plane. The positive direction is upward (so-called upper swing).
**Impact variables**				
	*θ*_*LOI*_	Line of impact angle		Angle between the line of impact (common normal to the surfaces in contact during impact) and the horizontal plane. The positive direction is contact with the lower half of the ball.
	*θ*_*undercut*_	Undercut angle		Angle between the bat velocity vector immediately before impact and the line of impact. The positive direction is when the bat velocity vector passes below the center of the ball.
	*θ*_*H*/*bat*_	Horizontal bat angle		Angle of orientation between the bat’s long axis projected onto the horizontal plane at impact and the x-axis (right and left directions). The positive direction is when the impacting surface of the bat directed towards the left field (pull side).
	*θ*_*V*/*bat*_	Vertical bat angle		Angle of orientation between the bat’s long axis at impact and the horizontal plane. The positive direction is when the barrel side of the bat directed downwards.

To evaluate the bat velocity and the ball–bat interaction, first, the midpoints of both ends of the bat were calculated by the coordinates of the eight markers attached to the bat. Although the ball–bat contact time is ~1 ms [25, 26], the positional information at impact cannot always be described accurately at a sampling frequency of 500 Hz as that used in this study. A previous study has also argued the sampling frequency and data accuracy [27]. Therefore, the 3D coordinates of the ball and bat for the 10 frames immediately before the impact were approximated as cubic functions and were derived as up-sampled filtering data at 4000 Hz, by using the regression equation. The moment of impact was defined as that point in time when the distance between the ball-bat contact surface was minimized using extrapolated coordinates; the mean value of this distance was found to be 2.1 ± 1.2 mm. The ball and bat velocities (speed and direction) immediately before and after the impact were calculated as the instantaneous velocity of one frame (1/4000 s) using a regression equation approximated as a cubic function. The horizontal launch angle of the batted ball (*θ*_*H*/*ball*_) is shown as the horizontal angle from the center line (y-axis), the opposite field being positive (Fig 3). The vertical launch angle of the batted ball (*θ*_*V*/*ball*_) is shown as the angle from the horizontal plane, which was 0°, with the upward trajectory being positive.

We defined the coordinate system fixed in the bat to calculate the ball location relative to the bat at ball impact (Fig 3). The vector from the center of the proximal end to the center of the distal end was defined as the x′-axis. The vector that is orthogonal to the x′-axis and parallel to the horizontal plane was defined as the y′-axis, and the cross product of the x′- and y′-axes was defined as the z′-axis. The angle representing the ball position relative to the bat along the bat’s short axis component at impact was defined as the line of impact angle (*θ*_*LOI*_). Furthermore, the upward/downward angle of the bat velocity vector (*θ*_*upward*_) was defined as the angle from the horizontal plane of the bat path immediately before impact. Undercut angle (*θ*_*undercut*_) was defined as the angle from the line of impact to the bat path immediately before impact on the vertical plane. The direction of the bat’s long axis was defined as the horizontal bat angle (*θ*_*H*/*bat*_) on the horizontal plane and the vertical bat angle (*θ*_*V*/*bat*_) on the vertical plane. To examine whether the bat speed immediately before the impact has the potential to act as an additional frictional force on the ball, the velocity of the x′-component at the bat’s hitting location (bat’s longitudinal speed: *v*_*x*′*/imp*_) was calculated.

**Fig 4:**
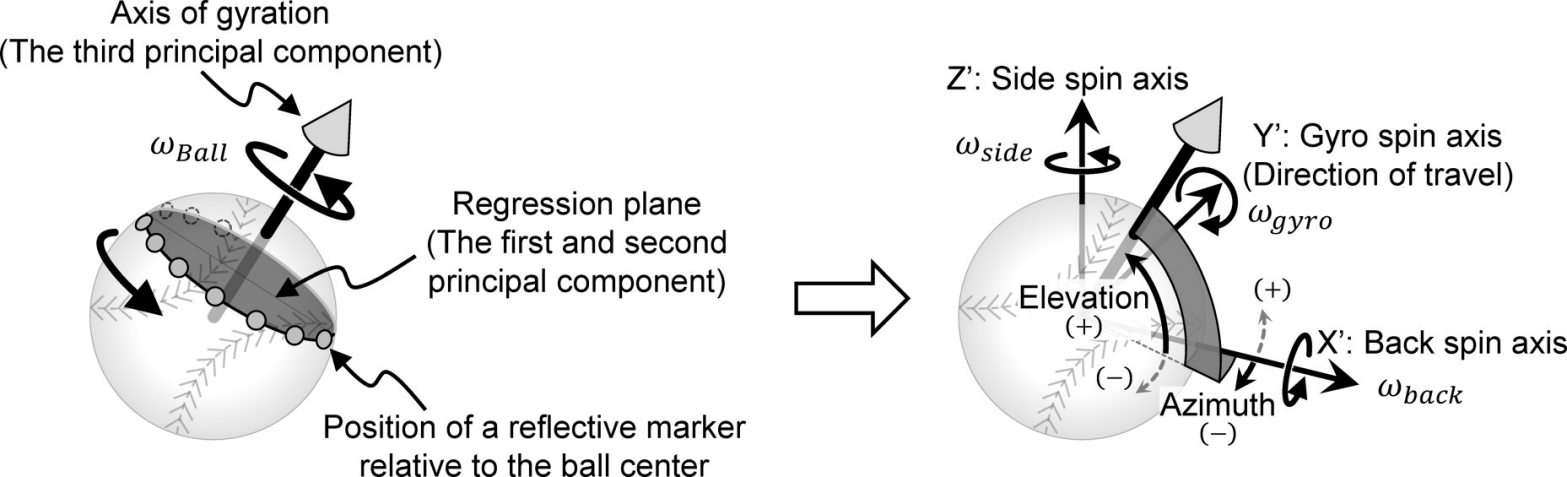
Calculation procedure of spin axis and spin rate of the batted ball.

The ball angular velocity (spin rate and spin axis) was derived using the coordinates of the ball center and those of a marker at one point on the ball surface (Fig 4). This point on the ball surface, which was selected for analyzing the ball spin, satisfied the following conditions: 1) the point was tracked 20 frames in a row; 2) it was the farthest from the center of rotation. The number of frames that the motion capture system was able to continuously track a marker that is required to calculate the ball spin was 20 to 30 frames. Therefore, the analytical range was set at 20 frames after impact. First, we calculated the relative coordinates of one point on the ball surface with respect to the ball center, and then calculated an approximate plane of the relative coordinates by principal component analysis. Then, we calculated the normal vector (third principal component) that is orthogonal to this plane. This normal vector represents the direction of the spin axis. This vector was converted in azimuth and elevation angle with respect to the ball trajectory (initial speed). The angular velocity was calculated as the angular displacement of 20 frames divided by the time spent. Further, the angular velocity of the batted ball was resolved into a back spin component, side spin component, and gyro spin component.

The direction of the batted ball was categorized into 10 horizontal and vertical sections based on the launch angle. In the horizontal direction, groups were separated in increments of 20°, resulting in five groups (−40°, −20°, 0°, 20°, 40°): −50° to −30° (−40°), −30° to −10° (−20°), −10° to 10° (0°), 10° to 30° (20°), and 30° to 50° (40°). Among the 602 hitting attempts that were included, 14 hitting attempts recorded a horizontal angle larger than ±50°. For the batted balls that landed on the foul ground, the accurate representative values (average) that were required for statistical analysis could not be obtained due to the small sample size. In addition, even if an apparent foul ball was included in one of the groups of launch direction, it was difficult to obtain the meaningful conclusions and the practical applications because it did not align with the primary purpose of this research. Therefore, the 14 hitting attempts that were recorded a horizontal angle larger than ±50° were excluded from the analysis. The vertical direction group was defined as the upward angle at which the spin axis of the batted ball shifts from top spin to back spin. The angle at which the spin axis switches was calculated using the linear regression method derived from the back spin (top spin) rate and the vertical launch angle of the batted ball. Batted balls at an angle larger than the derived turning point were categorized into the fly ball group (F), and those at a smaller angle into the ground ball group (G). Categorizing the horizontal and vertical hitting direction into 10 groups results in a loss of information of continuous data in the hitting direction. In addition, the closer the data are to the boundary of each group, the further they are from the representative value of the corresponding group. Nevertheless, in order to prioritize providing simple and clear answers to the research questions, this study focused on the comparison of each variable between the groups.

### Statistical analyses

To examine whether each variable in the impact phase differed between the hitting directions, two-way factorial analyses of variance (2 [fly ball (F) / ground ball (G)] × 5 [horizontal launch groups (−40°, −20°, 0°, 20°, 40°)]) were performed. Bonferroni adjustments were used for the pairwise comparisons between the groups. The testing for difference between the factors was performed when testing for interaction was not significant. Further, the simple main effect analysis for all groups was performed when the testing for interaction was significant.

Stepwise multiple regression analysis, with the side spin rate as a dependent variable, was performed to extract factors relating the side spin rate. The following four independent variables were analyzed: undercut angle, horizontal bat angle, vertical bat angle, and bat’s longitudinal speed. The line of impact angle and upward angle were excluded from the independent variables because of their strong association with the undercut angle. A multicollinearity check was conducted for all the variables input in the model using the variance inflation factor (VIF). The acceptable standards of the VIF were set at 10.

Bivariate regression was performed to examine the association between the dependent variable (vertical launch angle) and independent variables (line of impact angle and undercut angle). In addition, curve regression analysis was used to examine the association between the dependent variables (batted ball angular velocity and ball exit velocity) and independent variables (line of impact angle and undercut angle).

In observing the ball−bat collision, previous studies considered that the holding of the bat by hand does not affect the rebound characteristics of the ball in terms of the collision time and propagation speed of vibration associated with impact [26, 28]. Based on this knowledge, all participant attempts were analyzed collectively as independent data, and each variable was described as mean ± SD. All statistical analyses were performed with Statistical Package for the Social Sciences (SPSS) version 24 (IBM Corp., Armonk, NY, USA), with a significance level of *p* < 0.05.

## Results

**Fig 5:**
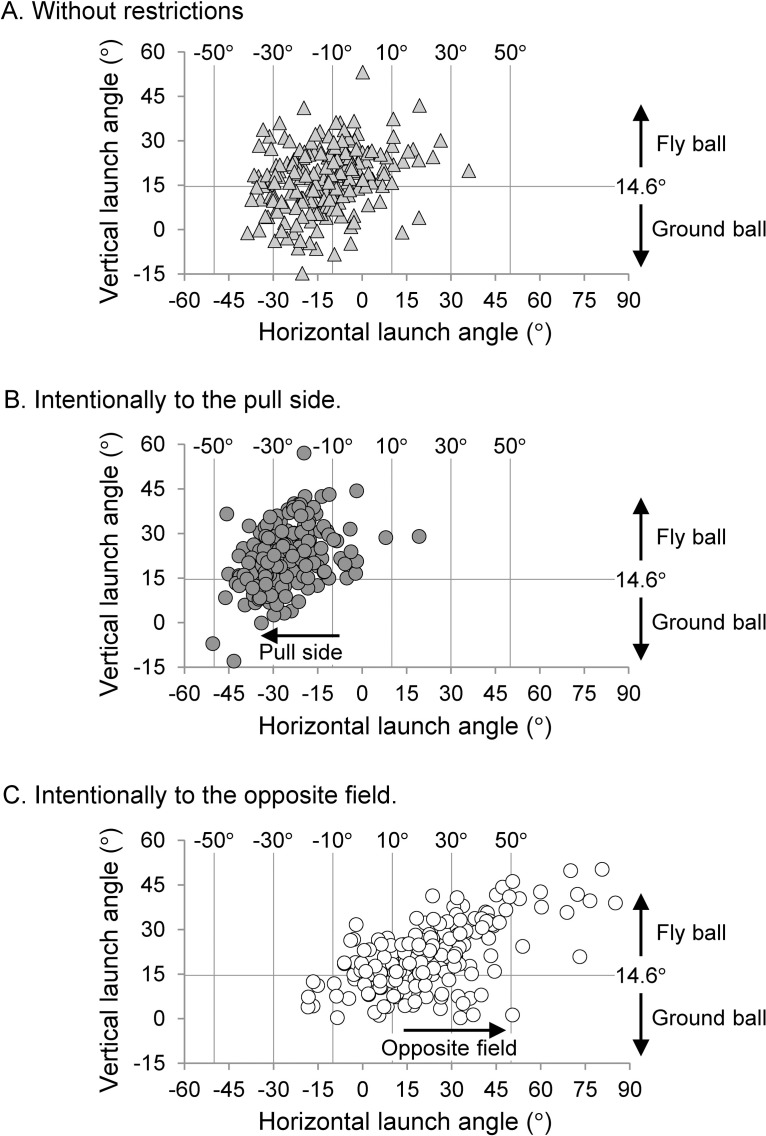
Distribution chart of launch direction for each hitting condition.

The bi-variate regression formula derived from the back spin rate (x) and vertical launch angle of the batted ball (y) was as follows: *y* = 0.958*x* − 14.03 (R^2^ = 0.721, *p* < 0.001). A 95% confidence interval for the correlation coefficient was 0.680–0.758. The vertical launch angle that would be the switching point between the top spin and back spin was 14.6°. The probability that the fly ball a contained topspin was 11.7% (= 50/374), and the probability that the ground ball contained a backspin was 25.0% (= 41/123). The number of trials for each launch group was 58 [F, −40°], 32 [G, −40°], 154 [F, −20°], 65 [G, −20°], 90 [F, 0°], 37 [G, 0°], 80 [F, 20°], 24 [G, 20°], 42 [F, 40°], and 6 [G, 40°]. Fig 5 shows the distribution of the batted balls’ launch direction for each hitting condition given to the participants. Both launch directions of the ground ball and fly ball were observed in all hitting conditions, and some batted balls were launched toward a direction different from that intended by the participants, especially in trials wherein the opposite field was intentionally selected.

**Fig 6:**
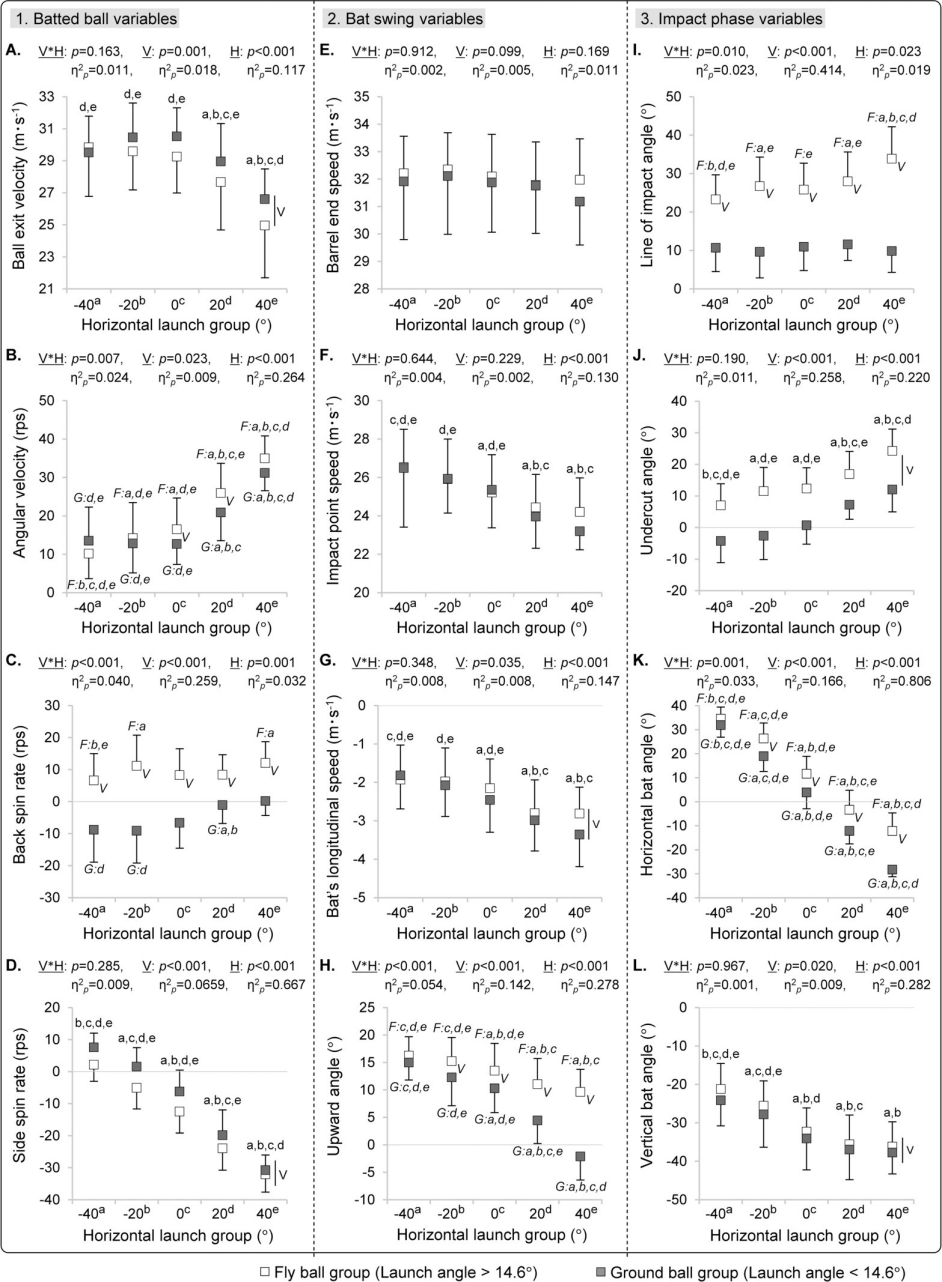
Mean and standard deviation of each variable for each group of the launch direction. V*H indicates the p-value and partial η^2^ of the vertical and horizontal interactions of the batted ball; V indicates the p-value, with partial η^2^ indicating the main effect of the vertical direction group and H indicating that of the horizontal direction group. Alphabets indicate significant differences between corresponding groups observed by the simple main effect analysis. Vertical bars indicate that interaction was absent, but a main effect existed between the vertical direction groups.

**Fig 7:**
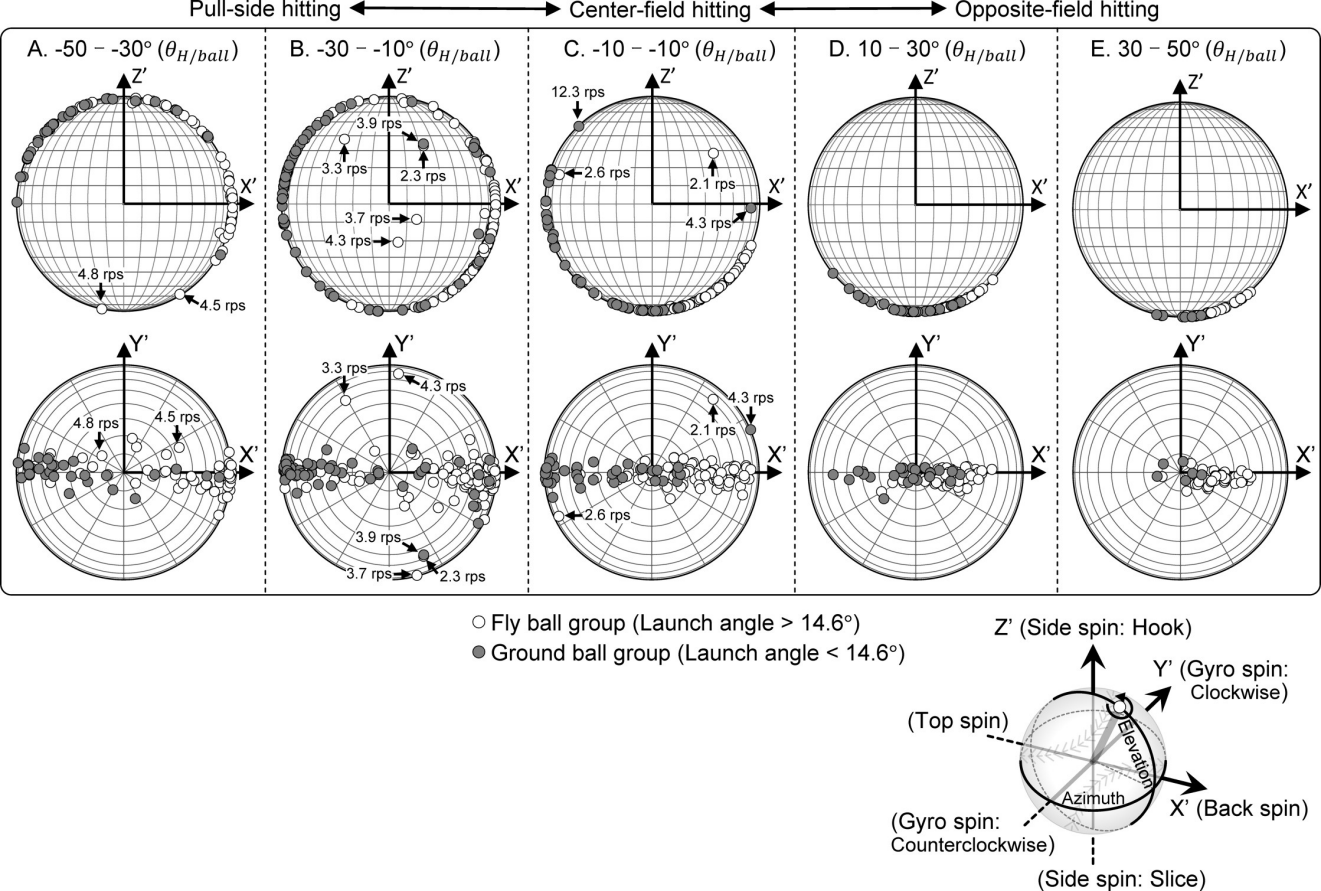
Direction of the spin axis of batted ball for each group sorted according to the horizontal launch direction. Each plot on the spherical surface represents the direction of the spin axis, and they are shown in two directions, with views seen from the near side (upper side) and from above (lower side) relative to the travel direction of the ball (y’-axis). The plot on the spherical surface does not display the front and back side of the sphere separately, and both are also shown on the front side. The spin rate of the batted ball is only represented in some plots that deviate from the overall trend.

Fig 6 compares the results of each variable according to the launch direction of the batted ball. The side spin rate differed significantly between almost all launch direction groups, and all batted balls toward the opposite field with a fly ball had a slice spin (*p* < 0.001, Fig 6D). The bat’s longitudinal speed toward the proximal side during opposite-field hitting was greater than that during pull-side hitting (*p* < 0.050, Fig 6G). The angle between the spin axis of the batted ball and the ball exit velocity vector was 91.1 ± 7.8° (95% CI: 90.8–92.1°, Fig 7), and no axis of rotation that generated a hook trajectory existed on the center and opposite field fly balls, aside from the line drive trajectories with low angular velocity (Fig 7C–7E).

All four independent variables (undercut angle: *θ*_*undercut*_, horizontal bat angle: *θ*_*H*/*bat*_, vertical bat angle: *θ*_*V*/*bat*_, and bat’s longitudinal speed: *v*_*x*′*/imp*_) were statistically identified as crucial factors contributing to the side spin rate (*ω*_*side*_) (R^2^ = 0.893, *p* < 0.001, *ω*_*side*_ = −0.727 × *θ*_*undercut*_ +0.305 × *θ*_*H*/*bat*_ +0.274 × *θ*_*V*/*bat*_ + 2.317 × *v*_*x*′*/bat*_ +6.497). The undercut angle contributed most to the side spin rate of the batted ball (ΔR^2^ = 0.560), followed by the horizontal bat angle (ΔR^2^ = 0.300). On the other hand, the vertical bat angle (ΔR^2^ = 0.017) and the bat’s longitudinal speed (ΔR^2^ = 0.017) only marginally affected the side spin rate. The variance inflation factor (VIF), which was a criterion of multicollinearity, was less than 10 for all independent variables (VIF (*θ*_*undercut*_) = 1.099, VIF (*θ*_*H*/*bat*_) = 1.860, VIF (*θ*_*V*/*bat*_) = 1.349, VIF (*v*_*x*′*/bat*_) = 1.528).

**Fig 8:**
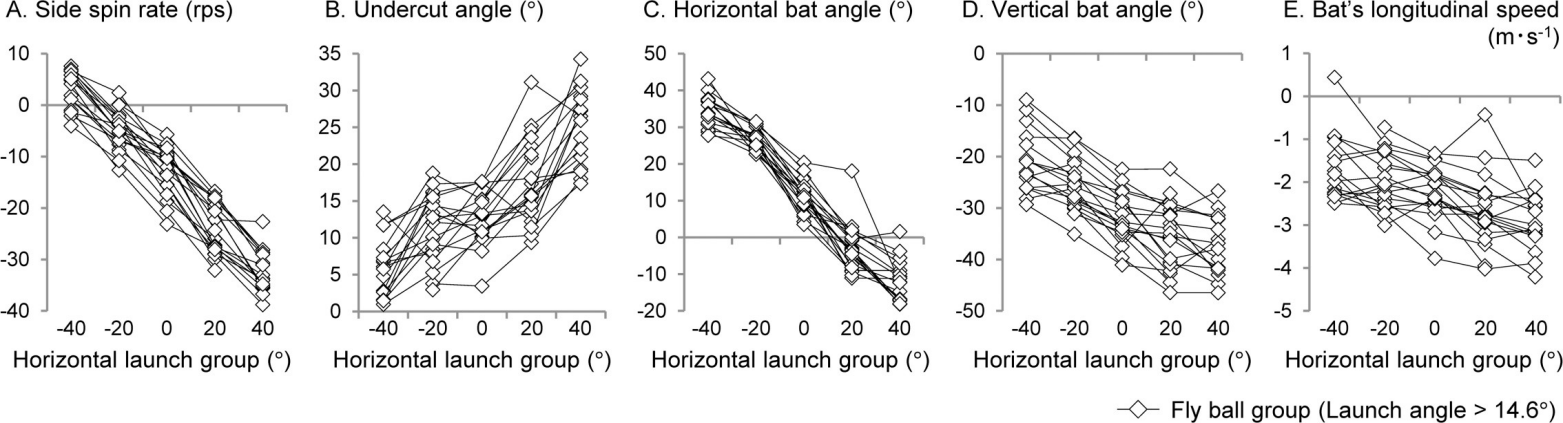
Changes in the mean value of all participants in the horizontal launch group used in the stepwise multiple regression analysis.

To verify whether the variation trend between the launch directions of each variable differs depending on the participant, variations in the mean values of all participants in the horizontal launch group of fly balls are shown in Fig 8. The data of all participants fluctuated according to the trends of two-way ANOVA results, with a few exceptions (Fig 6D, 6G, 6J–6L). However, even for the outliers that deviated from the overall trend, the reverse phenomenon of the side spin axis that the hook spin ball launched toward opposite field was not observed.

**Fig 9:**
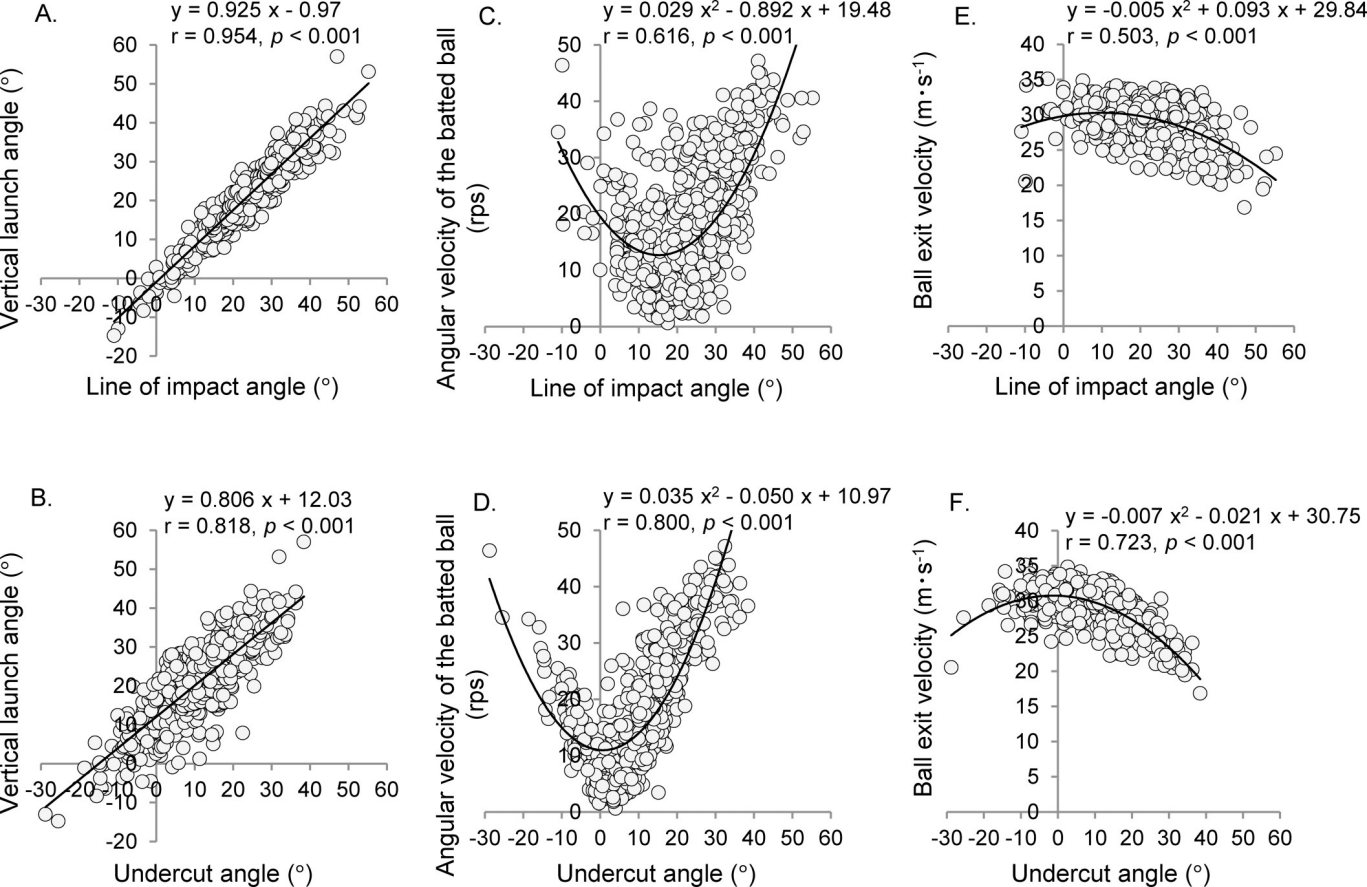
Association with the undercut and line of impact angles on the vertical launch angle, angular velocity of batted ball, and ball exit velocity. The horizontal axis in A, C, and E (top) indicates the line of the impact angle, whereas that in B, D, and F (bottom) indicates the undercut angle.

Fig 9 shows the relationship between the line of impact angle/undercut angle and the vertical angle of the batted ball, angular velocity of the batted ball, and ball exit velocity. The vertical launch angle was affected to a greater extent by the line of the impact angle (r = 0.954) than by the undercut angle (r = 0.818) (Fig 9A and 9B). However, the angular velocity of the batted ball and ball exit velocity were affected to a greater extent by the undercut angle (r = 0.800, *p* < 0.001; r = 0.723, *p* < 0.001, Fig 9D and 9F) than by the line of the impact angle (r = 0.616, *p* < 0.001; r = 0.503, *p* < 0.001, Fig 9C and 9E).

## Discussion

The purposes of this study were to clarify the association between the ball-bat interaction by the actual batters’ swing and launch direction of the batted ball in softball hitting. We hypothesized that ball−bat collision characteristics, including ball spin, are strongly associated with the launch direction. Therefore, a fly ball directed toward the opposite field includes slice spin regardless of the batter’s technique. This hypothesis was supported in this study.

Even if a ball was hit back toward the center field, the ball flew with slice spin because the bat’s distal side tilted downward at impact. The bat’s impact surface at the center-field hitting with fly ball was faced toward the pull side (11.6 ± 7.3° [F, 0°], Fig 6K). Furthermore, similar to previous findings in baseball [21–23], the distal side of the bat’s long axis was tilted downward at ball impact (32.3°, Fig 6L). With this downward tilt, the participants naturally contact the diagonally lower side of the ball (near side of the batter) when attempting to hit a fly ball. Therefore, we consider that the batted ball flying toward center field includes a slice spin because the slice spin caused by the undercut angle exceeded the hook spin element caused by the impact surface of the bat facing the pull side.

The bat does not rotate two-dimensionally on the horizontal plane during the swing, but accelerates in a three-dimensional trajectory including up-and-down movement. In other words, a fly ball hit back toward the center field flew as a trajectory that included slice spin and not a pure backspin, because the bat’s long axis tilted three-dimensionally at ball-bat impact. The bat’s longitudinal speed, which is one of the main interests of this study, statistically affected the side spin rate of the batted ball, but only to a small extent (ΔR^2^ = 0.017). The force acting on the bat in the forward and centripetal directions on the swing plane during the impact phase [29, 30] could be a factor generating longitudinal speed. However, the bat’s longitudinal speed in itself was only 9.0 ± 3.9% of the resultant speed. In addition, the behavior of the bat, including the longitudinal speed at impact, changed more remarkably because of the direction of the batted ball than because of the variation between participants or attempts (Figs 6 and 8). Therefore, because it is unlikely that the batter can control the batted ball’s spin independently without relation to launch direction, it would be extremely difficult to impart a hook spin to the batted ball when the batter hits it toward the opposite field.

The angular velocity of the batted ball had a higher correlation with the undercut angle than the ball’s impact location relative to the bat (line of the impact angle) (Fig 9C and 9D). The undercut angle includes information of the bat trajectory immediately before the impact (upward angle). This trajectory could have affected the angular velocity of the batted ball. Conversely, the line of the impact angle was highly related to the vertical launch angle than that of the undercut angle (Fig 9A and 9B). In other words, the fluctuation factor of the angular velocity is partially different from the vertical launch angle; even if the ball−bat positional relationship at impact is the same, the angular velocity tends to fluctuate depending on the bat trajectory immediately before impact. In this study, the threshold between the fly ball and ground ball was defined as the vertical launch angle (14.6°) at which the spin axis of the batted ball shifts from top spin to back spin. However, the spin axis was not completely reversed at the threshold; the probability that the fly ball contained topspin was 11.7%, and the probability that the ground ball contained backspin was 25.0%. It is considered that this between participant differences in the bat’s upward angle were the main factor of gap. Getting another perspective on this result, when applying the findings of the basic research obtained by collision experiments against a fixed bat or ball to actual hitting, it is necessary to interpret data on relative motion.

**Fig 10:**
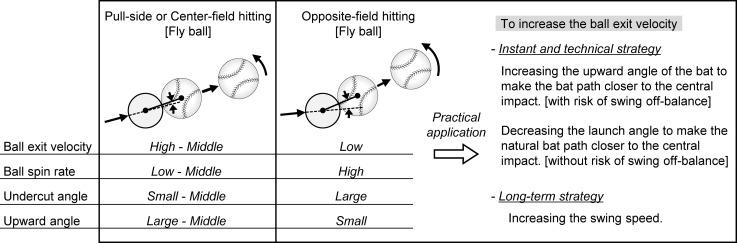
Practical application for improving the performance of the opposite-field hitting.

The larger undercut angle observed for opposite-field hitting was caused by the upward angle of the bat being smaller in opposite-field hitting (Fig 6H and 6J). Consequently, batted balls in opposite-field hitting had a larger spin rate. Furthermore, the absolute value of the vertical bat angle was larger with opposite-field hitting than with pull side hitting (Fig 6L). Therefore, the proportion of the side spin component tended to be high in opposite-field hitting, in addition to the large angular velocity. Based on the above discussion on the three elements (initial speed, launch angle, and spin) that represent the characteristics of a batted ball, the characteristics of a batted ball’s spin strongly associates with the flight direction—thus, it may be wise for the batter to focus only on the initial speed and launch angle. As a specific strategy, because minimizing the absolute value of the undercut angle and maximizing the bat speed would synergistically maximize the ball exit velocity (Fig 6A, 6F and 6J), the batter must increase the bat’s upward angle in cases of opposite-field hitting. Adaptable batters who are capable of freely changing the swing trajectory according to every intended launch direction can hit the ball with a high speed in any direction. However, the bat’s upward angle immediately before impact changes naturally with the hitting direction, and when hitting toward the opposite field, it becomes relatively downward compared to the pull side hitting (Fig 6H). Therefore, in order to generate the high ball exit velocity in the opposite field hitting, it is necessary to change the swing trajectory of the bat and provide an upward angle similar to that of the pull side hitting. The impact conditions for maximizing the flight distance and maximizing the batted ball speed are different, but this gap is particularly large in the case of opposite-field hitting. Because it is necessary to go against its own natural swing trajectory, this intentional change in swing trajectory can result in a risk of off-balance of the batter’s swing motion. Therefore, a higher swing speed than that of pull-side hitting will be required to carry a ball having a long flight distance in the opposite-field side. Considering these points, hitting the ball with a low trajectory according to the natural swing trajectory may help in improve the batting average of opposite-field hitting because of the increase in ball exit velocity (Fig 10).

**Fig 11:**
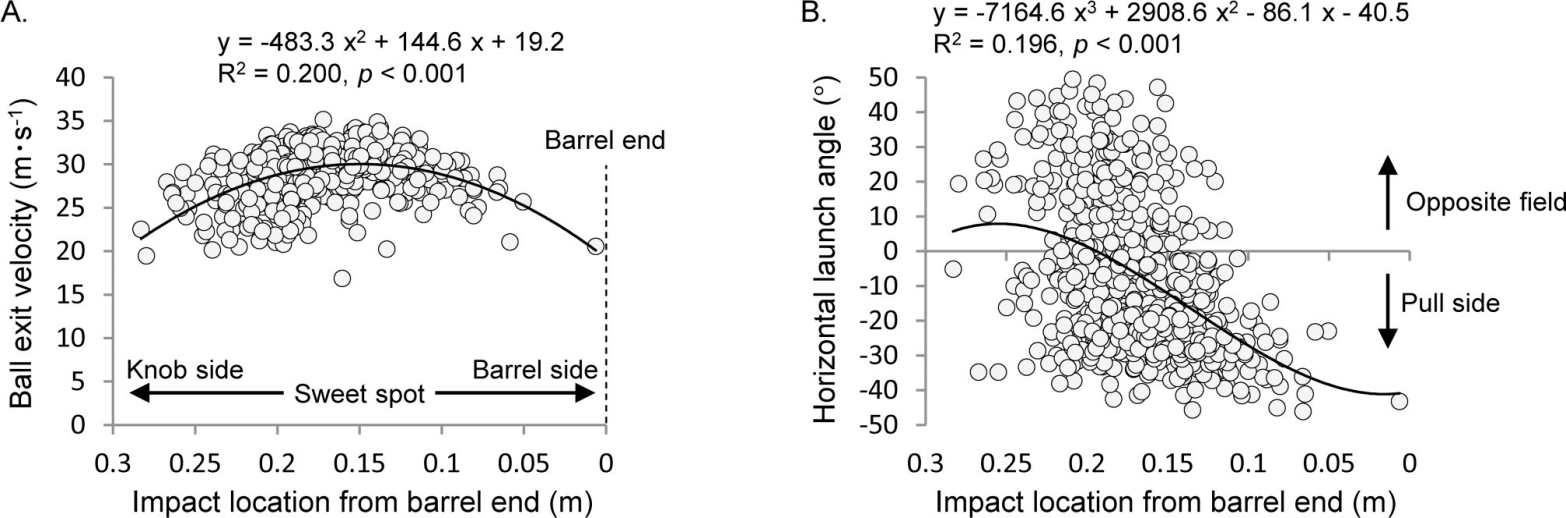
Association with the impact location from barrel end on the ball exit velocity and horizontal launch angle. Impact location indicates the ball coordinates along the bat’s long axis (x′-axis) relative to the coordinate system fixed in the bat. The positive and negative directions of x′-axis were reversed with an emphasis on clarity.

In addition, several fly balls that intentionally went toward the opposite field were scattered toward the foul ground (Fig 5C); the range of the launch direction was wider than that of the intentional pull-side hitting. The horizontal scattering of the batted ball is affected by both the timing control and the interaction with the bat’s 3D tilt at ball−bat impact (Fig 1). Because the bat’s downward tilt in opposite-field hitting was larger than that in pull-side hitting (Fig 6L), the oblique impact along the bat’s short axis could probably have greatly fluctuated the horizontal launch angle. Furthermore, although the barrel end speed did not differ significantly between the launch groups, the impact point speed in opposite-field hitting was smaller than that in pull-side hitting (Fig 6E and 6F). This suggests that the impact location along the long axis of the bat differs depending on the hitting direction, and the relationship between the impact location and the horizontal launch angle is shown in Fig 11 for verification. The ball exit velocity was maximized at a position of 15.0 cm from the barrel end of the bat (Fig 11A). There were many attempts to hit the ball on the knob side rather than the sweet spot (Fig 11B) in the opposite-field hitting. By contrast, the wide distribution around the position of the sweet spot was observed in the pull-side hitting. Thus, it is assumed that the participants tried to hit the ball at a position close to the own body in opposite-field hitting to delay the ball−bat impact timing. A mishit with respect to each hitting direction can be caused by many factors. It is important to ensure that the batter focuses on hitting the ball close to the most optimal point on the bat’s surface (both along the long and short axes) for any pitch.

### Limitations of the study

This study has several limitations. First, pitches were conducted by a pitching machine. Some studies have indicated that visual information about the pitching form until ball release is an important element for the batter to control the timing of impact [31, 32]. Although this study did not analyze timing control by batters, timing errors from using a machine may have affected the kinematic characteristics of the bat. It was a limitation of this study, but the pitched ball speed was slow, and thus, even if the visual information until the ball release was insufficient, the participant could afford to adjust their swing practically against the thrown ball.

Second, the speed of the pitched ball was much slower than the speed of the ball thrown by elite softball pitchers in games. The ball’s release speed pitched by Olympic windmill pitchers (27 ± 2 m·s^-1^) [33] will probably decelerate to about 24–25 m/s by the time it reaches the home plate, but this speed is to a large extent different from than the speed in this study (9.6 ± 0.2 m·s^-1^). This particular concern is associated with the influence on the rebound characteristics of the batted ball and the batter’s swing motion. When impact variables other than bat swing speed (*v*_*bat*_) and pitched ball speed (*v*_*pitch*_) are constant, the batted ball speed (*v*_*ball*_) is derived by *v*_*ball*_ = (1 + *e*_*A*_) *v*_*bat*_ + *e*_*A*_ *v*_*pitch*_ [34]; *e*_*A*_ denotes the apparent coefficient of restitution [28], and is ~0.2 when the ball hits the sweet spot of the bat [34]. Therefore, the pitched ball speed affects the batted ball speed, but not as much as the bat swing speed. Similarly, variations in the pitched ball speed are considered to have a certain effect on the angular velocity of the batted ball. The effect of the pitched ball speed on the batter’s swing characteristics cannot be evaluated because the swing in a real game or during high-speed ball practice has not been investigated. It is not known whether the participants were able to execute the same swing as in the actual game because even expert softball players hit balls at slow speeds in regular practice; however, they were able to execute the swing with maximum effort and no motion constraints. The strength of this study lies in demonstrating the impact techniques resulting from the actual swing by elite players according to the batted ball direction. The above discussion indicates that the slow pitched ball speed possibly effects the numerical value of results uniformly, but the conclusion on the research question of this study is considered to be independent of the pitched ball speed. Nevertheless, further research involving simulations of more practical and real game-like conditions would help improve the reliability of our findings and improve athlete performance.

Third, although the data for all participants was analyzed as independent of each other, the data for within- and between-participant variations have been combined. The reason for choosing this method of analysis is based on the findings of a previous study [26, 28], which states that the characteristics of the batted ball is unaffected by the strength of hand grip. Although there were differences in the numerical value of each variable of each hitting direction between participants, the data of all participants fluctuated in a similar tendency as the statistical result (two-way ANOVA), with a few exceptions (Fig 8). In addition, the scatter plots (Figs 9 and 11), wherein all participants all data were plotted as independent samples, were theoretically explained the relationship between variables. Therefore, although the statistical results include the influence of the participants, this variable factor probably does not distort the conclusion for research question. Investigating the actual swing movement of elite players is especially good for understanding the range of variations in the batted ball that can be controlled by the batter’s skill.

## Conclusions

We compared the ball−bat interaction in different directions of the batted ball during softball hitting, and identified factors relating the characteristics of the batted ball. The distal side of the bat tilted downward at ball−bat impact in all trials. Therefore, a ball that was hit at the diagonally lower side of the ball (near side of the batter) launched with a spin that included a slice component, even if the bat was directed toward the center field. The bat’s upward path in opposite-field hitting was lower than that in pull-side hitting, increasing the ball angular velocity and decreasing ball exit velocity in hitting opposite-field fly ball because the element of oblique impact at ball-bat collision increases. Therefore, to hit a ball back toward the opposite field with a high ball exit velocity, the batter should aim at a lower launch angle than that in pull-side hitting or the bat’s path should be directed upward more than natural own swing trajectory. In this study, the use of the ball delivery machine and the speed of the slower pitched ball were the limiting factors in the generalization of the findings.

## Supporting information

S1 Table Numerical value for each launch group of the variables related to batted ball, bat swing, and impact phase. (XLSX)

S2 Table The raw data of one participant representing the coordinates of reflective markers attached to the ball and bat. (XLSX)
